# Safety Climate Perceptions in the Construction Industry of Saudi Arabia: The Current Situation

**DOI:** 10.3390/ijerph17186717

**Published:** 2020-09-15

**Authors:** Ibrahim Mosly, Anas A. Makki

**Affiliations:** 1 Department of Civil Engineering, Faculty of Engineering—Rabigh Branch, King Abdulaziz University, Jeddah 21589, Saudi Arabia; 2 Department of Industrial Engineering, Faculty of Engineering—Rabigh Branch, King Abdulaziz University, Jeddah 21589, Saudi Arabia; nhmakki@kau.edu.sa

**Keywords:** safety climate, perception, construction, factors, Saudi Arabia

## Abstract

Workers’ wellbeing and safety is important in the construction industry due to the high risk of accidents. Safety climate development is a positive initial step toward raising the safety levels of construction practitioners. This study aims at revealing the factors influencing safety climate perceptions in the construction industry of Saudi Arabia. A set of extracted factors from the literature was validated and used to design a comprehensive questionnaire survey. Data was collected from 401 personnel working on 3 large construction project sites in Saudi Arabia. Descriptive statistics and the crosstabulation algorithm, Kendall’s tau-b correlation test, were used to analyze the data. The study revealed a set of 13 factors influencing safety climate perceptions, which are: Supervision, guidance and inspection, appraisal of risks and hazards, social security and health insurance, workmate influences, management safety justice, management commitment to safety, education and training, communication, workers’ safety commitment, workers’ attitude toward health and safety, workers’ involvement, supportive environment, and competence. The results also indicate the significant and anticipated role of top management in safety climate at sites. Implications of this study include assisting construction industry stakeholders to better understand and enhance safety climate, which in turn will lead to improved safety behavior, culture, motivation, and performance.

## 1. Introduction

Safety involves people’s wellbeing and lives, making its implementation essential in construction projects [1]. Workers in the construction industry are in need of safety measures assimilation. The construction industry is a dangerous one, with high levels of injuries and death ratios compared to other industries [2]. Records show that the number of nonfatal occupational injuries in the U.S. construction industry in the year 2018 was 199,100 cases [3]. Furthermore, in the U.K., the average annual number of nonfatal occupational injuries in the construction industry from 2016–2019 was 54,000 cases [4]. Moreover, in Saudi Arabia, the construction industry accounted for the highest number of injuries among other industries with 16,968 cases in the year 2018 [5]. The significance of this industry cannot be overstated, as it delivers the infrastructure needed by the other industries and reflects the country’s economic development level [6]. Disastrous safety accidents may occur if there is poor recognition of hazards and safety risks are underestimated [7]. Being a vital industry with high levels of safety risks, stakeholders’ attention should focus on determining robust solutions. The lack of hazard recognition and the underestimation of safety risk are both widespread in the construction industry [7]. Workers in the construction industry experience high rates of injury and illness compared to those in other industries [8]. Training and research are tools that can assist in converting the situation of this industry. A large number of workers perceive production as a priority compared to managers, who have a better overall safety culture appraisal, and this may indicate that workers could take more risks to meet deadlines and expose themselves to injuries and illnesses [8]. Focus on raising safety awareness should start with the workers, who represent the weakest and most physically affected group in this industry. The widespread hiring of subcontractors in the construction industry has led to an increase in major safety concerns among general contractors, researchers, and health and safety organizations [9]. This emphasizes the need to conduct safety research to enhance safety levels at construction sites. Areas for safety enhancement can be identified through safety climate measurement [10]. Safety climate describes the perception, attitudes, and beliefs of workers on risk and safety and is mostly measured through questionnaire surveys to provide an idea on the current safety situation [11]. This makes safety climate an important initial intervention tool. An organization’s safety climate should be measured regularly, as it will assist competent safety personnel in identifying and enhancing the policies, procedures, and practices of that organization [12]. Currently, it has become necessary for construction entities to embrace construction workers to assure a solid commitment to implementing safety policies and plans [13]. Safety participation and compliance significantly influence safety climate [12]. The training of competent safety teams with competent leaders at construction sites will have a positive influence on its safety. Safety leadership endorses constructive safety climate development, and this may result in improved construction safety [1].

The significance of safety climate is observed by its influence on safety behavior, safety motivation, safety culture, and safety performance. Firstly, there is an association between safety behavior and safety motivation. Several accidents are caused by unsafe actions, and this indicates that worker behavior is considered as an extremely significant workplace safety factor [14]. A study conducted in Saudi Arabia to examine the relationships among safety motivation, safety climate, safety behavior, and safety outcomes reported the following results: 1. Safety behavior can be positively influenced by safety motivation because of the safety climate in the organization; 2. safety behavior might forecast safety outcomes [15]. Another study in Australia has validated the significant role of supervisors’ leadership and communication approach in creating a positive safety climate and influencing their workers’ health and safety behaviors [16]. Once again, through supervisors’ motivational communication, a positive safety climate can be created and as a result workers’ safety behavior can be changed. Secondly, to further enhance safety climate, it is vital for an organization to have a positive safety culture, which also influences safety performance. Management should build communication channels and support workers with a positive organizational culture as well as ensure that their workers are protected, regardless of their background or employment type [17]. High-level supervisors have a significant role in facilitating the link between organizational safety climate and safety performance [18]. This means that an organization’s team leader play an important role in safety climate. A study claims that project managers’ skills will support the execution of targeted safety management tasks [1]. The application of these tasks can endorse safety climate, thus improving safety performance and eliminating unsafe situations along with preventing accidents [1]. Furthermore, increasing evidence shows that safety culture directly affects safety performance [8].

Research can influence the development and promotion of safety climate in the construction industry. One study emphasized the significance of enlightening positive safety climate, as it will lead to the enhancement of hazard recognition and safety risk perception [7]. Construction workers perceive risk perception as a job hindrance [19]. An example of a job hindrance is work pressure, and this causes a negative impact on both safety motivation and behavior [19]. Safety climate assists in changing workers’ risk perception from a job hindrance to a job challenge [19]. Employees of companies with high levels of safety climate behave safely, and they perceive that they are encouraged to retain good safety practices in spite of high production pressure [20]. A study in Taiwan concluded that the safety climate perception of management is higher than that of laborers and that this difference can be used to improve strategies for enhancing safety climate on construction sites [17]. Improving the overall construction safety levels is the responsibility of construction managers, as they enhance safety perception by safety training and better practices [21]. Furthermore, better safety climate perception can be achieved when there is clear communication on rules [22]. Here, research studies emphasized the important role of team leaders and their means of communication with their teams to deliver safety climate.

Due to the high number of accidents in the construction industry of Saudi Arabia [6,23,24,25], several approaches should be investigated to enhance safety levels at Saudi Arabian construction sites, including safety climate. Currently, there is a lack of research in the area of safety climate in the construction industry of Saudi Arabia. Only a limited number of research studies explored safety climate in the construction industry of Saudi Arabia, including the studies done by Panuwatwanich et al. [15], Erogul and Alyami [26], Sanni-Anibire et al. [27], and Al-Haadir et al. [28]. Thus, there is a lack of research in the area of safety climate in the construction industry of Saudi Arabia, which necessitates its exploration in a comprehensive manner, with focus on identifying the factors influencing safety climate as a first step. This is because increasing empirical evidences links the improvement in safety climate with the improvement in construction site safety practices [29]. Furthermore, several studies found that safety climate is an important predictor of safety performance [30]. Nevertheless, a significant factor that expresses safety climate perception is the workers’ perception of workplace hazards and risks, which is related to their safety climate perception of their company and their industry [31]. There is an association between the employees’ safety climate perception and the safety of their workplace [32,33,34,35]. Furthermore, a number of project specific characteristics can influence the relation between safety climate and safety culture such as, project duration, project size, and contractor grade [36]. This could indicate that changing the employees’ workplace may cause a change in their perception on safety climate depending on the level of safety of the workplace. Therefore, the evaluation of safety climate perception should be done based on the employees’ risk perception as well as for their overall workplace safety climate perception.

This study will investigate the factors influencing safety climate in the construction industry based on the perception of industry practitioners. Furthermore, it will highlight the significant factors that in turn assist management level stakeholders in enhancing the safety levels of their organizations, leading to improved safety performance at construction sites.

## 2. Materials and Methods

A case study approach and quantitative research design was followed to achieve the objectives of this study. A set of 18 factors influencing safety climate in the construction industry was adopted from Mosly [2]. In their original literature review, the author only included articles from the Web of Science platform journal list. The search engine of the Web of Science platform is the most powerful and is used by many researchers to produce high-quality research [37]. A further extensive literature review of previous studies was made in the present study to verify and further support the 18 factor set adopted from Mosly [2] in a comprehensive manner that included all related studies from all available journals in the Saudi Digital Library (SDL). The SDL provides access to more than 300 global publishers, including Elsevier, Springer, Pearson, Wiley, Taylor & Francis, McGraw-Hill, Yell University, Oxford University Press, and Harvard University Press [38]. The key words used in the search included “construction”, “safety climate”, and “factors”. Additional references were added to the previous literature review by Mosly [2] compiling a total of 62 references used to identify the 18 factors influencing safety climate in the construction industry. This was made to enhance the reference list used to identify the influencing factors and make it more comprehensive. Those factors are broadly pertaining to the safety commitment, safety interaction, and safety support aspects of safety climate from both the management and workers’ sides. Subsequently, the extracted set of factors was used to design a questionnaire survey by which detailed and comprehensive data were collected. The collected data included employee socio-demographic characteristics, ratings of factors influencing safety climate, and their overall rating of the project site safety climate.

Employees were asked about their sociodemographic characteristics, including age, nationality, education level, occupation, trade specialty, and years of experience. Further, on a 5-point Likert scale (i.e., extremely important, important, neither, unimportant, extremely unimportant), they were asked to rate the importance of the extracted factors in influencing safety climate in their construction project sites. Finally, on a 5-point scale (i.e., excellent, good, neither, poor, extremely poor), they were also asked to evaluate the overall safety climate in their project sites.

Following a random sampling approach, cross-sectional data were collected in the period starting from July 2019 to January 2020 from site employees of 3 available, typically large (i.e., worth 140 million USD) construction projects in Saudi Arabia (see Table 1 for key safety parameters of sample projects). A random sample of 500 employees working at the 3 construction sites of ongoing projects was targeted. Four hundred and one employees responded and successfully completed the questionnaire, representing a response rate of 80.2%. Data was collected via site visits, face-to-face interviews, and Google Forms for ensuring a single response from each respondent, recording the collected data, and initial database set-up. All construction site employees of all types, levels and backgrounds had an equal probability of participating in the survey and being in the sample. Ethical approval from the ethical committee at the Center of Excellence in Genomic Medicine Research, King Abdulaziz University, was obtained for conducting this research questionnaire survey. (HA-02-J003)

The Statistical Package for the Social Sciences SPSS version 23.0 (SPSS Inc.; Chicago, IL, USA) [39], was used to analyze the collected data. Descriptive statistics was used to analyze the sociodemographic characteristics of the respondents, and their perceived ratings on the safety climate influencing factors. Additionally, as the collected data were ordinal, the crosstabulation algorithm, Kendall’s tau-b correlation test [40,41] was used to reveal the statistically significant influencing factors that act as predominant determinants of the perceived overall safety climate evaluation of construction sites. Kendall’s tau-b is a non-parametric test used to measure the ordinal correlation between variables based on pairs of ranked data representing the level of concordance between them. Therefore, the correlation coefficients using [39] and in accordance to [41] were calculated using the following algorithm (Equations (1)–(7)):

Let,

*X_i_*: Distinct values of row variable arranged in ascending order (*X*_1_ < *X*_2_ < … < *X_R_*),
*Y_j_*: Distinct values of column variable arranged in ascending order (*Y*_1_ < *Y*_2_ < … < *Y_C_*),
*f_ij_*: Sum of cell weights for cases in cell (*i*,*j*),
*c_j_*: $\sum_{i\;=\;1}^{R}f_{ij}$, the *j*th column subtotal,
*r_i_*: $\sum_{j\;=\;1}^{C}f_{ij}$, the *i*th row subtotal,
*W*: $\sum_{j=1}^{C}c_{j\;}=\;\sum_{i=1}^{R}r_{i}$, the grand total,
  (1) $$D_{r}=W^{2}-\overset{R}{\underset{i=1}{\sum }}r_{i}^{2},$$
  (2) $$D_{c}=W^{2}-\overset{C}{\underset{j=1}{\sum }}c_{j}^{2},$$
  (3) $$C_{ij}=\underset{h<i}{\sum }\underset{k<j}{\sum }f_{hk}+\underset{h>i}{\sum }\underset{k>j}{\sum }f_{hk},$$
  (4) $$D_{ij}=\underset{h<i}{\sum }\underset{k>j}{\sum }f_{hk}+\underset{h>i}{\sum }\underset{k<j}{\sum }f_{hk},$$
  (5) $$P=\underset{i,j}{\sum }f_{ij}\;C_{ij},$$
  (6) $$Q=\underset{i,j}{\sum }f_{ij}\;D_{ij},$$
  where in SPSS,
*P*: is double the number of concordant pairs.
*Q*: is double the number of discordant pairs.
*D_r_*: is double *P* + *Q* + *X*_0_ (the number of concordant pairs, discordant pairs, and pairs on which the row variable is tied).
*D_c_*: is double *P* + *Q* + *Y*_0_ (the number of concordant pairs, discordant pairs, and pairs on which the column variable is tied).
accordingly, Kendall’s Tau-b correlation coefficient is given by Equation (7):
  (7) $$\tau_{b}=\frac{P\;-\;Q}{\sqrt{D_{r}\;D_{c}}}$$

The results of the analysis along with a discussion are presented subsequently.

## 3. Results and Discussion

The resulting set of extracted and verified safety climate factors from the extensive literature review process is listed in Table 2 including their supporting references, and a brief description of each of them is presented in Table 3. The most mentioned influencing factor in the literature review with respect to safety climate in the construction industry was management commitment to safety. This emphasizes the important role of management in construction site safety and safety climate effect. Moreover, the factors communication, supervision, guidance and inspection, education and training, and safety rules, and procedures, were also highly mentioned in related studies. It also indicated their major impact on construction industry safety and safety climate. The least mentioned safety climate factor was social security and health insurance, as it was only mentioned in one study.

Table 4 presents the descriptive statistics of the collected sample of employee’s sociodemographic characteristics. This includes the sample size and percentage of each sociodemographic subgroup in the collected sample. The demographics included: Age, nationality, education, occupation, years of experience, and trade specialty. The descriptive statistics presented in Table 4 show that the surveyed construction employees in the sample are distributed among eight age categories, and most of them were aged between 30 and 40 years. Furthermore, they were mainly from Pakistan, Egypt, Yemen, India, and Syria, respectively. The respondents’ education level ranged from illiterates to bachelor’s degree, with the largest subgroup being employees with secondary education level. Among six construction industry occupations, workers represented a large subgroup of the sample. Moreover, most of the employees in the sample demonstrated a work experience ranging from new employees to employees with 11 to 15 years of experience. Lastly, among 14 construction industry specialties, carpenters represented the largest portion of the used sample in this study. The sociodemographic subgroup sample sizes and percentages are considered representative of many typical construction project sites in Saudi Arabia.

The employees in the collected sample were asked to rate, on a 5-point scale, the importance of the 18 extracted factors influencing safety climate to their construction project site. Table 5 presents the descriptive statistics of their ratings, including the distribution of the sample size and percentage of each level of importance for each influencing factor. Furthermore, it includes the overall mean and standard deviation along with the rank and level of each factor. After ranking the factors based on their mean, the factors supervision and guidance, education and training, and management commitment to safety, were ranked, in order, as the top three influencing factors on safety climate in construction project sites of Saudi Arabia. Moreover, these factors received a rating level of “extremely important” based on their mean scores. This indicates the important role of the top management in facilitating safety climate perception in employees. This can be accomplished by providing specialized safety supervision and guidance through appointing competent safety personnel for construction site auditing and inspection, as well as offering continued safety guidance. Supervisors have significantly higher safety climate perception compared to workers [96]. They conduct a significant role in providing workers with needed safety support through ongoing interaction, such as demonstrating how to perform tasks safely as well as guaranteeing that rewards are provided for accomplishing safety targets on site [97]. Due to work pressure and higher standard of safety performance, supervisors are more subjected to psychological stress than workers [96]. Automatic computer vision-based techniques can assist supervisors in their daily tasks and reduce the amount of stress that they face. For instance, a study has tested and validated the use of automatic computer vision-based techniques to monitor workers crowdedness for the purpose of proactive safety management [98]. This assisted in addressing the issues caused by high crowdedness that includes hazardous working environments, negative workers’ behavior, lack of concern for safety climate, and low productivity [98]. Moreover, employees should be offered safety training and workshops at all levels, along with the distribution of safety educational material. Theoretical and practical training programs are important in improving safety behavior [99]. It is necessary to provide less experienced workers with sufficient safety training, as more experienced workers are cautious toward hazards due to their safety knowledge [100]. Building information modelling applications that focus on safety education and training can also augment safety climate [101]. Also, new training methods such as safety training park can be adopted to develop safety climate within organizations [102]. Strong commitment to safety measures by management will require sufficient financial and moral support. A study specified the optimal safety investment to be 1% of the total project value [103]. Furthermore, a study has identified the safety climate factor of safety management rules and regulations as the most effective safety factor [104]. As stated previously in the introduction section, organizations’ safety policies, procedures, and practices must be measured regularly, which reflects management commitment to safety. Generally, the development, fund, and implementation of policies should be comprehensive with local to national scale interdisciplinary efforts [105]. The remaining influencing factors on safety climate received a rating level of “important” based on their ranked mean scores. Based on the overall weighted mean of the 18 factors, the overall rating level of all factors in the questionnaire was “important”, indicating an overall agreement of the surveyed construction site employees on the importance of the 18 factors in influencing the safety climate at construction sites in Saudi Arabia. Given that workers represent the largest subgroup in the sample (i.e., 73.3%) (Table 4) and from the ranking of factors in the questionnaire (Table 5), it is apparent that workers indicate the extremely important role of management and the anticipated top-down direction of safety assimilation on construction sites for a better safety climate from their perspective. Although the safety climate factor social security and health insurance was only mentioned in one reference in the literature review (see Table 2), the study participants ranked it in fourth place, giving it high priority among the influencing safety climate factors in the context of the Saudi Arabian construction industry. The participants viewed social security and health insurance as a significant element for attaining safety climate. Despite that the communication safety climate factor was ranked 17 out of the 18 influencing factors, it was still important. This might be due to the fact that most workers in the construction industry of Saudi Arabia are expatriates of diverse nationalities (see Table 4) and they see communication as a major obstacle and an important safety climate element. Supervisors play a vital role in building safety climate through safety communication within a group of workers, which in turn changes workers’ safety compliance and behaviors [106]. Synergistic benefits can be achieved by positive safety climate and maintaining high levels of safety communication [107]. In fact, a study resulted in confirming that the link between safety climate and safety outcomes is totally arbitrated by safety communication [108]. An approach such as the lean project delivery system can be used to enhance positive safety climate in the project, as it can integrate stakeholder teams and improve communications [109]. Also, information and communication technology systems can assist in improving safety and quality [110]. A pilot study that investigated the use of information and communication technology systems in the form of a mobile application presented an enhancement of 30% in quality and a reduction of 90% in unsafe behaviors [110].

As mentioned earlier, the same construction site employees were also asked to evaluate the overall safety climate in their construction project site on a 5-point scale (i.e., excellent, good, neither, poor, extremely poor). Table 6 provides descriptive statistics of their perceived evaluations, including the distribution of the sample size and percentage of each evaluation level of safety climate on their construction site. It also shows the overall mean and standard deviation along with the overall evaluation level. Table 6 shows that the overall safety climate level on construction sites where the surveyed employees work is “good”, based on the mean of their evaluations. A limited number of participants perceived the safety climate levels of their construction sites to “poor” or “extremely poor”.

Subsequently, to reveal the statistically significant influencing factors that act as predominant determinants of the perceived overall safety climate evaluation of construction sites, Kendall’s tau-b correlation test was conducted between the perceived overall safety climate evaluation of construction sites (Table 6) and the perceived importance of each of the 18 factors influencing safety climate (Table 5). The results of the analysis, presented in Table 7, show the statistically significant correlations between the factors influencing safety climate perceived importance and the perceived overall safety climate evaluation. Significant factors are ranked based on their Kendall’s tau-b values and their associated level of statistical significance. Thirteen out of 18 influencing factors on safety climate were significant predominant determinants of perceived safety climate evaluation at their construction sites. The factors were supervision and guidance, appraisal of risks and hazards, social security and health insurance, workmate influences, management safety justice, management commitment to safety, education and training, and communication, demonstrating a statistical significance level of *p* < 0.001. Whereas the factors: Workers safety commitment, workers’ attitude toward health and safety, workers’ involvement, supportive environment, and competence demonstrated statistical significance levels of *p* < 0.01. We noted that most of the influencing factors demonstrating *p*-values of <0.001 are related to the top management. On the other hand, we also noted that most of the influencing factors demonstrating *p*-values <0.01 are related to the workers. This indicates the importance of the support provided by management to construction site employees in many forms, the role of management commitment to safety, and then safety aspects pertaining to workers in determining safety climate level on construction sites. Table 7 presents the significant factors influencing safety climate in the construction industry of Saudi Arabia.

Construction industry stakeholders should focus in-depth on these factors to raise safety climate levels among their employees. This will lead to raising the safety perception at construction sites and as a result increase the safety levels by reducing the rate of accidents. Furthermore, these factors represent the datum for future research in safety climate in the construction industry of Saudi Arabia. More studies could be developed based on these influencing factors.

## 4. Conclusions

In this study, the aim was to reveal factors influencing safety climate in the construction industry of Saudi Arabia from the industry practitioner’s perspective. The study has revealed a total of 13 statistically significant influencing factors that act as predominant determinants of the overall safety climate evaluation of construction sites. These influencing factors are: Supervision and guidance, appraisal of risks and hazards, social security and health insurance, workmate influences, management safety justice, management commitment to safety, education and training, communication, workers’ safety commitment, workers’ attitude toward health and safety, workers’ involvement, supportive environment, and competence. The results of this study indicate the significant and anticipated role of top management in the construction industry of Saudi Arabia to play in safety climate from construction site employees’ point of view. This also provides further evidence and emphasis on the safety climate literature on the need for the application of a top-down safety climate assimilation approach. Furthermore, despite the low number of previous studies considering social security and health insurance as an important influencing factor of safety climate, our study reveals the significance of this factor in influencing safety climate in the construction industry of Saudi Arabia. This finding provides evidence for the construction industry practitioners’ perceived significance of social security and health insurance, which could be attributed to the environment of the construction industry being project-based and a high safety risk industry.

The results of this study will assist stakeholders of the construction industry of Saudi Arabia to better understand and enhance safety climate in their organizations, which in turn will lead to the development of safety performance on construction sites. This better understanding can be reached using the revealed set of factors with their perceived importance describing the current situation in construction sites. Furthermore, the revealed correlations of the set of influencing factors with perceived safety climate evaluations in construction sites from the viewpoint of their employees, assist in determining areas of improvement to enhance safety climate. Moreover, the presented statistically significant factors influencing safety climate in the construction industry of Saudi Arabia can be used in many future research directions such as safety climate, safety performance, and safety leadership in the construction industry context, to mention just a few.

It is recommended for organizations involved in the construction industry of Saudi Arabia to increase the emphasis on enhancing safety climate perception on their sites. This could be attained through different means, including the adoption and implementation of novel techniques and systems that support supervision and guidance, offering of extensive training programs, and the development of encouraging safety policies, procedures, and practices that demonstrate the management commitment to safety.

Results of this study are limited to representing patterns within the collected data (July 2019 to January 2020) from employees working on 3 large construction sites in Saudi Arabia. Despite that the 3 large construction sites are deemed representative of typical construction sites in Saudi Arabia, reconducting the study and collecting data from other construction sites at a different temporal context will further confirm the results of this study. Moreover, despite the fairly large sample size used in this study (i.e., *n* = 401), conducting the study using a larger sample size is another future research direction to confirm the revealed set of influencing factors of safety climate. Furthermore, the next step to this work is to categories or cluster the revealed set of influencing factors using a dimension reduction statistical approach to reveal the main latent components of safety climate perceptions in construction sites. Moreover, studying the safety climate perceptions of different sociodemographic groups of construction site employees to reveal the influence of sociodemographic characteristics on safety climate perceptions is also a future research direction. Finally, developing a safety climate prediction model using the revealed set of factors in this study that can predict perception levels of construction site employees is another future research direction.

## Figures and Tables

**Table 1:** Key safety parameters of sample projects.

Safety Parameters	Project 1 ($97 million)	Project 2 ($26 million)	Project 3 ($27 million)
Number of Accidents Occurred	24	20	11
Written Safety Policy	√	√	X
Availability of Competent Safety Teams	√	√	√
Availability of Safety Instructions and Guidelines	√	√	√
Availability of Personnel Protective Equipment	√	√	√
Provision of Safety Education and Training for Employees	√	√	√
Implementation of Safety Procedures	√	√	√

**Table 2:** List of extracted factors influencing safety climate at construction project sites from the previous literature.

Safety Climate Factor *	References	Number of References
Management Commitment to Safety	[10,12,15,18,29,30,32,42,43,44,45,46,47,48,49,50,51,52,53,54,55,56,57,58,59,60,61,62,63,64,65,66,67,68,69,70,71,72,73,74,75,76,77,78]	44
Communication	[12,15,30,42,43,44,46,51,54,55,58,59,60,61,62,63,64,69,76,77,79,80,81,82,83,84,85,86,87]	29
Supervision, Guidance, and Inspection	[18,29,32,43,44,46,47,49,50,53,55,57,58,61,63,65,67,71,72,74,75,80,88,89,90,91]	26
Education and Training	[12,32,44,46,47,52,56,57,58,60,61,62,69,73,75,76,77,82,83,86,92,93,94]	23
Safety Rules, and Procedures	[10,15,32,36,42,43,44,45,46,47,48,49,53,55,57,58,60,61,69,73,76,79,86]	23
Co-worker Influence	[12,18,29,30,32,42,47,48,50,51,64,71,72,75,79,80,89,90,95]	19
Workers’ Involvement	[10,12,29,30,32,43,45,46,48,50,55,56,58,60,61,62,71]	17
Work Pressure and Intensity	[12,15,43,49,55,56,58,60,61,66,67,69,73,79,80]	15
Worker’s Attitude toward Health and Safety	[10,12,30,43,47,48,50,51,69,73,76,81]	12
Supportive Environment	[43,50,51,55,56,57,60,76,84]	9
Safety Resources	[12,30,32,42,54,57,79]	7
Appraisal of Risk and Hazard	[15,32,43,54,55,58]	6
Competence	[12,15,32,43,51,55]	6
Workers’ Commitment to Safety	[29,51,54,59,84]	5
Adequacy of Safety Procedures	[36,68,70,79,83]	5
Management Safety Justice	[51,54,59,84]	4
Safety Value and Reward System	[53,80,94]	3
Social Security and Health Insurance	[80]	1

* Adopted and enhanced from Mosly [2].

**Table 3:** Description of safety climate factors.

Safety Climate Factor	Description	References
Management Commitment to Safety	The priority level and care that management dedicate to workers’ safety.	[12,15,43]
Communication	Effective communication by management and workers’ feedback.	[12,15,43]
Supervision, Guidance, and Inspection	Assistance and assurance that a safety program is fully implemented on site.	[18,29,43]
Education and Training	Information, instructions, and learning materials provided to workers on safety.	[58,60,93]
Safety Rules, and Procedures	Safety rules and procedures set by the organization.	[12,43,58]
Co-worker Influence	The influence of co-worker’s perceptions/practices on each other in terms of safety aspects.	[12,29,42]
Workers’ Involvement	Involvement and contribution of workers in safety activities.	[12,15,43]
Work Pressure and Intensity	The level at which workers feel pressurized to complete a task within a specific deadline.	[15,43,58]
Worker’s Attitude toward Health and Safety	The perception of the worker towards aspects of health and safety, and the willingness degree to risk taking.	[12,43,47]
Supportive Environment	An overall safety trust and support between a group of employees.	[43,50,76]
Safety Resources	Sufficient safety resources allocated by management for the safe conduct of tasks at site.	[12,32,42]
Appraisal of Risk and Hazard	The ability to assess risks and hazards.	[32,43,55]
Competence	The general background of workers’ knowledge, training, qualification, and skills.	[15,43,55]
Workers’ Commitment to Safety	The priority level and care that workers dedicate to their own and others safety on site.	[51]
Adequacy of Safety Procedures	Content of safety procedures being clear, comprehensive, correct, and reflect actual safety needs.	[70,83]
Management Safety Justice	The degree of quality to which the management can deal fairly with workers involved in safety accidents.	[51]
Safety Value and Reward System	Incentives to encourage workers on safe practices and good behavior.	[80]
Social Security and Health Insurance	Providing workers with legal contracts and medical insurance.	[80]

**Table 4:** Research sample sociodemographic (*n* = 401).

Characteristic	Groups	*n*	%	Characteristic	Groups	*n*	%
Age (years)	18–20	2	0.50	Occupation	Worker	294	73.31
	21–25	51	12.72		Technician	15	3.74
	26–30	57	14.20		Supervisor	50	12.50
	31–35	89	22.20		Architect	3	0.75
	36–40	105	26.20		Engineer	29	7.20
	41–45	65	16.20		Manager	10	2.50
	46–50	23	5.74	-	-	-	-
	More than 50	9	2.24	Experience (years)	0–5	108	26.93
-	-	-	-		6–10	134	33.41
Nationality	Sudanese	3	0.75		11–15	107	26.70
	Philippine	3	0.75		16–20	35	8.73
	Bangladeshi	6	1.50		More than 20	17	4.23
	Somali	7	1.75	-	-	-	-
	Syrian	50	12.47	Trade specialty	Carpenter	100	24.93
	Indian	55	13.71		Blacksmith	82	20.45
	Yemeni	58	14.50		Bricklaying	39	9.72
	Egyptian	90	22.40		Painter	5	1.25
	Pakistani	129	32.17		Plumbing	34	8.50
-					Cement and concrete	31	7.73
Education	Illiterate	70	17.45		Crane operator	7	1.75
	Elementary	68	16.96		Surveyor	9	2.24
	Intermediate	73	18.20		Mechanical	4	1.00
	Secondary	99	24.69		Architecture	3	0.75
	Diploma	31	7.70		Electrical	9	2.24
	Bachelor	60	15.00		Administration	15	3.74
-	-	-	-		Civil	50	12.50
-	-	-	-		Safety and quality control	13	3.20

**Table 5:** Descriptive statistics of factors influencing safety climate (*n* = 401).

Factor		Level					Mean	Standard Deviation		Rank	Level ^a^
		5	4	3	2	1					
Supervision, guidance, and inspection	*n*	204	127	63	7	0	4.32	0.798		1	5
	%	50.9	31.7	15.7	1.7	0.0					
Education and training	*n*	206	129	53	11	2	4.31	0.840		2	5
	%	51.4	32.2	13.2	2.7	0.5					
Management commitment to safety	*n*	186	133	68	12	2	4.22	0.867		3	5
	%	46.4	33.2	17.0	3.0	0.5					
Social security and health insurance	*n*	184	132	66	16	3	4.19	0.903		4	4
	%	45.9	32.9	16.5	4.0	0.7					
Appraisal of risks and hazards	*n*	148	167	68	18	0	4.11	0.841		5	4
	%	36.9	41.6	17.0	4.5	0.0					
Competence	*n*	142	168	71	15	5	4.06	0.889		6	4
	%	35.4	41.9	17.7	3.7	1.2					
Workers’ safety commitment	*n*	146	145	83	24	3	4.01	0.938		7	4
	%	36.4	36.2	20.7	6.0	0.7					
Management safety justice	*n*	149	127	95	23	7	3.97	0.998		8	4
	%	37.2	31.7	23.7	5.7	1.7					
Safety resources	*n*	146	136	79	31	9	3.95	1.035		9	4
	%	36.4	33.9	19.7	7.7	2.2					
Work pressure and intensity	*n*	87	173	104	32	5	3.76	0.923		10	4
	%	21.7	43.1	25.9	8.0	1.2					
Supportive environment	*n*	76	185	109	29	2	3.76	0.860		11	4
	%	19.0	46.1	27.2	7.2	0.5					
Safety rules and procedures	*n*	103	143	103	42	10	3.72	1.039		12	4
	%	25.7	35.7	25.7	10.5	2.5					
Safety value and reward system	*n*	80	148	133	40	0	3.67	0.907		13	4
	%	20.0	36.9	33.2	10.0	0.0					
Adequacy of procedures	*n*	70	141	128	46	16	3.51	1.035		14	4
	%	17.5	35.2	31.9	11.5	4.0					
Workers’ involvement	*n*	53	146	148	50	4	3.48	0.908		15	4
	%	13.2	36.4	36.9	12.5	1.0					
Workmate influences	*n*	60	133	136	67	5	3.44	0.978		6	4
	%	15.0	33.2	33.9	16.7	1.2					
Communication	*n*	52	145	135	64	5	3.44	0.950		17	4
	%	13.0	36.2	33.7	16.0	1.2					
Workers’ attitude toward health and safety	*n*	28	159	170	42	2	3.42	0.790		18	4
	%	7.0	39.7	42.4	10.5	0.5					
Weighted Mean							3.85		Overall Level		4
Standard Deviation								0.43			

Level: 5 = Extremely important; 4 = important; 3 = neither; 2 = unimportant; 1 = extremely unimportant. ^a^ Level based on a score interval mean on an equal interval lengths of 0.8: 5 = (4.20, 5), 4 = (3.40, 4.20), 3 = (2.60, 3.40), 2 = (1.80:2.60), 1 = (1:1.80).

**Table 6:** Descriptive statistics of perceived overall safety climate ratings (*n* = 401).

**Safety Climate Ratings**		**Level**					**Mean**	**Standard Deviation**	**Overall** **Level ^a^**
		**5**	**4**	**3**	**2**	**1**			
	*n*	65	202	103	29	2	3.75	0.831	4
	%	16.21	50.37	25.69	7.23	0.50			

Level: 5 = Excellent; 4 = good; 3 = neither; 2 = poor; 1 = extremely poor. **^a^** Overall level based on a score interval mean on an equal interval lengths of 0.8: 5 = (4.20, 5), 4 = (3.40, 4.20), 3 = (2.60, 3.40), 2 = (1.80:2.60), 1 = (1:1.80).

**Table 7:** Statistically significant correlations between the perceived importance of factors influencing safety climate and the perceived overall safety climate ratings.

Factors Influencing Safety Climate in the Construction Industry of Saudi Arabia	Kendall’s tau-b
Supervision and guidance	0.208 ***
Appraisal of risks and hazards	0.196 ***
Social security and health insurance	0.191 ***
Workmate influences	0.180 ***
Management safety justice	0.181 ***
Management commitment to safety	0.173 ***
Education and training	0.166 ***
Communication	0.160 ***
Workers’ safety commitment	0.144 **
Workers’ attitude toward health and safety	0.143 **
Workers’ involvement	0.120 **
Supportive environment	0.125 **
Competence	0.123 **

** *p* < 0.01, *** *p* < 0.001.
